# How does air quality affect the health of children and adolescents?

**DOI:** 10.1016/j.jped.2024.11.009

**Published:** 2025-01-03

**Authors:** Herberto José Chong-Neto, Nelson Augusto Rosário Filho

**Affiliations:** Department of Pediatrics, Federal University of Paraná, Curitiba, Paraná, Brazil

**Keywords:** Air pollution, Child health, Adolescents

## Abstract

**Objectives:**

To assess how air quality and pollutants affect the health of children and adolescents.

**Source of data:**

A narrative review of recent literature was conducted using PubMed databases, focusing on studies published between 2015 and 2023. The keywords included “air pollution”, “child health”, “adolescents”, “respiratory diseases” and “cognitive development”. The studies were selected based on their relevance to the pediatric community and impacts on air quality, emphasizing original peer-reviewed research and meta-analyses.

**Synthesis of data:**

Exposure to pollutants in the air during the formative and development years can lead to respiratory disorders, neurodevelopmental impairment, and exacerbated chronic conditions. This review synthesizes current evidence on the relationship between air quality and pediatric health, emphasizing the effects of specific pollutants, mechanisms of harm, and long-term implications.

**Conclusions:**

From respiratory disorders to neurodevelopmental problems, air pollution, remains a widespread threat, particularly to vulnerable populations. Immediate actions at the political, community, individual, and industry levels are necessary to mitigate these risks.

## Introduction

Air pollution is one of the prominent factors of adverse health effects, affecting not only the respiratory tract, which is exposed to the highest concentrations of pollutants throughout life but almost all organs of the body.^1^ Noncommunicable diseases (NCDs) increasingly dominate the global impact of human health, causing 41 million deaths each year (74 % of all deaths). Of these, 77 % occur in low- and middle-income regions, which are the least prepared for them.^1^

A high proportion of NCDs are inflammatory and immune-mediated (IMNCDs), including common diseases such as arterial hypertension, allergies, autoimmune diseases, type 1 diabetes, and dementia, for which there is no permanent cure.^2^

Current evidence incriminates the social and environmental determinants of health for the onset of these disorders by promoting gene-environmental interactions exacerbated by the effects of climate change.^3^

The global pollution crisis continues to have a negative impact on human health. In asthma, for instance, environmental pollution can interact with genetic variants to increase the risk of the disease.^4^

A gene-versus-environment (GxE) study in mice demonstrated that the magnitude of airway hyperreactivity in response to particles from combustion engines depends on the genotypes at the Dapp1 locus.^5^ In humans, genome-wide association studies (GWAS) identified a GxE interaction between airway hyperreactivity caused by diesel combustion and a locus on chromosome 3 encoding DAPP1.^5^

Low-income countries bear a disproportionately high burden of global morbidity and mortality caused by chronic respiratory diseases, including asthma, chronic obstructive pulmonary disease, bronchiectasis, and post-tuberculosis pulmonary sequelae. These are strongly associated with poverty, infectious diseases, and other non-communicable diseases and contribute to complex multimorbidity, with adverse consequences for the lives and livelihoods of those affected.^6^

The population inequality within the countries' own borders is a matter of concern for health authorities. For instance, the prevalence of asthma is higher among low-income African-American children, who are more likely to reside near highways and industrial zones. Health disparity in asthma can therefore be partially attributed to the fact that exposure to pollution disproportionately affects low-income populations. As currently known genetic and environmental risk factors cannot fully explain asthma risk, there is a great need to further delineate gene and environment interactions.^7^ NCDs are caused and exacerbated by climate change. Lived experiences of individuals affected by NCDs, including preventable deaths among children caused by air pollution, poor housing, and allergies, offer a powerful approach to encouraging and driving environmental policy changes.^7^

Evidence-driven, the World Health Organization (WHO) has lowered its health-related limit values for particulate matter (PM)_2.5_ and nitrogen dioxide (NO_2_) air pollution. Currently, 97 % of the urban population of the European Union is exposed to emissions that exceed these limits, and this is associated with up to 5 million premature deaths/year.^1^

## Types of pollutants

Exposure occurs not only through external air pollution but also by indoor pollution and indoor environments such as schools, daycare centers, and workplaces, where part of the day is spent. Children suffer more from the consequences of exposure to air pollutants because they are in the growth and development phase.^8^

Children are especially susceptible to air pollution due to:

• Immature respiratory systems: increased ventilation rates and partially developed lung defenses.
• Higher exposure levels: relative to body weight, children inhale more air compared to adults.
• Developmental plasticity: critical periods of organ and neuron development increase the deleterious effect of toxic exposures.
• Behavioral patterns: outdoor play increases exposure to environmental pollutants.

Domestic pollution involves biological agents, such as dust mite allergens, insects, pollen, animal hair, fungi, bacterial endotoxins, chemical substances from cleaning materials, detergents, and insecticides. Outdoor pollutants also contribute to household pollution.^8^

Non-biological household pollutants are gases, particulate matter, formaldehyde, and volatile organic compounds (VOCs). Household air pollution resulting from the burning of polluting fuels such as kerosene and biomass is a global environmental health problem, especially in developing countries. Secondhand smoke has been widely studied and also contributes to the development of chronic non-communicable diseases.^8^

A pilot study analyzed the environmental health of children living in urban and rural areas of Uruguaiana, Brazil. The study was carried out using a questionnaire applied to parents or guardians of children treated at the Children's Polyclinic in that city, between January and October 2021. Children living in rural areas had higher exposure to pesticides (32.7 %), chemicals (32.7 %), proximity to crops (74.5 %) and sources of contamination (32.7 %). They also had more contact with animals (87.3 %) and less sanitation and garbage collection infrastructure. Children living in urban areas were more exposed to vehicular traffic (85 %) and air pollution. The environmental history is crucial to identify harmful exposures in the environment where children live, play, and study.^9^

Lifestyle and exposure to pollutants, both biological and non-biological, modify the host's and environment's microbiome, causing an immune imbalance with inflammatory consequences and the development of diseases.^10^

The impact of particulate pollutants on human health is not only caused by the direct effects but may also involve the effect on the bacterial behavior of the host. Carbon, the main component of particulate matter (PM), is implicated in the predisposition to infectious respiratory diseases, inducing changes in bacterial biofilms *of Streptococcus pneumoniae* and *Staphylococcus aureus*.^10^, ^11^, ^12^

## Respiratory diseases

Genetic predisposition combined with environmental exposure to inhaled substances that affect the airways is the strongest risk factor for developing asthma. In recent years, robust epidemiological evidence has shown that air pollution not only affects patients with preexisting asthma but can also act to initiate it.^13^ Moreover, a given individual submitted to the set of all exposures in the external environment from preconception onwards will suffer the consequences of exposures at the cell and organic level.^14^

The impacts of exposure to air pollution during the prenatal period can affect organogenesis and organ development, which can lead to long-term complications, affecting respiratory health in different ways.^15^^,^^16^

Recent studies have shown the accumulation of black carbon on the fetal side of the placenta, suggesting that environmental particles can be transported to the fetus and represent a potential mechanism that may explain the detrimental effects of pollution from early life.^17^

Exposure during pregnancy (24–36 wk) to 2 mg/m^3^ or more of PM_2.5_ in ambient air during the saccular phase of lung development was associated with a 1.29-fold increased risk of asthma (95 % CI: 1.06–1.58), current asthma (RR: 1.27; 95 % CI: 1.04–1.54), but no current wheezing.^17^^,^^18^ Impaired lung development contributes to infant mortality in individuals exposed to this environmental condition.^15^, ^16^, ^17^, ^18^, ^19^, ^20^

Exposure to PM_10_ from heavy road traffic during pregnancy was associated with significant reductions in lung function.^16^ Maternal exposure to traffic-related NO_2_, especially in the first trimester of pregnancy, has been associated with an increased risk of developing asthma and rhinitis in children.^21^

The components of cigarette smoke are potentially toxic to the fetus, including lead, nicotine, cotinine, cyanide, cadmium, mercury, CO, and polycyclic aromatic hydrocarbon (PAH). CO reduces the supply of O_2_ to the fetus, leading to hypoxia, as it binds to hemoglobin with an affinity 200-fold greater than O_2_ and hinders or releases O_2_ to cells. Chronic mild hypoxia of fetal tissue may persist for five to six hours after the mother stops inhaling cigarette smoke.^22^

A recent meta-analysis evaluated the deleterious effects of exposure to tobacco smoke during pregnancy, associated with harmful effects on the fetus and newborn in the first two years of life.^23^ Regarding the respiratory system, exposure during pregnancy and passive exposure after delivery has been associated with an increased risk of wheezing in children under two years of age, higher frequency of respiratory tract infections in children under two years of age (including bronchiolitis, pneumonia, bronchitis, pulmonary tuberculosis, otitis media), and increased risk of developing asthma.^23^ It is believed that at the epigenetic level, exposure to tobacco smoke during pregnancy can alter DNA methylation and messenger RNA expression in placental tissue, which can determine changes in gene expression that affect the development of health conditions in offspring.^24^

Exposure to PM has been associated with impaired lung function in children, documented by decreased peak expiratory flow rates and forced expiratory volume in one second, especially in children with asthma, and clinically externalized by the increased number of exacerbations, emergency room visits, hospitalizations, and childhood deaths.^25^^,^^26^

Exposure to PM_10_ and NO_2_ has been associated with reduced eosinophilic and neutrophilic inflammation of the respiratory mucosa in children without wheezing. On the other hand, long-term exposure to PM_10_ has been associated with eosinophilic inflammation in children with wheezing, suggesting that it may contribute to the development of asthma, and inflammation, and promote airway remodeling.^27^ Secondhand exposure to tobacco smoke or nicotine-releasing devices has been associated with an increased risk of wheezing and asthma in children.^23^

## Cardiovascular impairment

Studies show that exposure to TRAP (Traffic-Related Air Pollution) during childhood and adolescence can negatively impact cardiovascular health. For example, TRAP exposure was significantly correlated with elevated blood pressure (BP) in children,^28^ rapid weight gain, or higher body mass index (BMI)^29^^,^^30^ and was associated with increased acute morbidity and mortality from cardiovascular disease (CVD).^31^ Finally, a systematic review found that when children and adolescents exercised in highly polluted areas, the reported benefits of BP on cardiopulmonary fitness were nil, and even had detrimental health effects due to breathing polluted air, such as a decrease in glucose resistance and increased risk of developing asthma.^32^

Short- and long-term exposure to PM contributes significantly to cardiovascular toxicity and increased risk of developing CVD. Studies have reported a significant association between PM_2.5_ exposure and elevated blood pressure (BP) in children,^33^, ^34^, ^35^ which is also affected by short- and long-term PM_10_ exposure.^33^^,^^34^ Long-term exposure to PM_10_ is associated with an increased risk of hypertension (34), as well as exposure to PM_2.5_,^35^ which they all associated with a higher likelihood of childhood obesity and increased BMI.^36^^,^^37^

Short- and long-term childhood exposure to NO_2_ is associated with elevated BP^38^ and increased prevalence or risk of hypertension in children and adolescents^35^ and is significantly associated with risk of childhood obesity and higher BMI.^38^ Specifically, the odds increased by 12 % (95 % CI: 1.06–1.18) when one is obese and exposed to higher concentrations of NO_2_ than in less exposed children.

Long-term exposure to ozone (O_3_) is significantly and positively associated with high blood pressure,^33^ and 10 μg/m^3^ increases in O_3_ exposure are associated with an increased risk of obesity.^37^ Sanders et al. found significant associations between exposure to lead, inorganic arsenic, and cadmium and arterial hypertension in childhood.^28^

## Cognitive, mental, and behavioral health

Evidence shows that exposure to TRAP-related air pollution can damage the developing brain and central nervous system (CNS) in a number of ways.^39^ Specifically, exposure to TRAP is associated with impaired mental and/or psychomotor development,^40^ behavioral disorders, prevalence and development of autism spectrum disorder (ASD),^39^ decreased cognitive function, and increased neuroinflammatory markers.^39^^,^^41^^,^^42^ Additionally, two systematic reviews suggest that exposure to air pollution is associated with changes in brain structure, function, and metabolism;^43^ however, future studies are needed to confirm them.^44^ Short- and long-term TRAP exposure in and around school and at home are significantly associated with lower academic achievement scores,^42^^,^^45^ impairment in problem-solving skills, lower grade point average (GPA), and will negatively affect executive function, with the effect becoming more severe over time of exposure.^46^, ^47^, ^48^

Regarding mental health, articles suggest that adolescent exposure to TRAP was significantly associated with symptoms of depression,^43^ generalized anxiety disorder, psychotic disorders, delusions, hallucinations, unusual experiences, and poorer overall mental health.^42^^,^^48^^,^^49^

Exposure to PM was associated with an increased risk of childhood ASD^50^ and an increased risk of attention deficit hyperactivity disorder (ADHD),^51^ with a more significant effect in boys than in girls.^50^ Moreover, other reviews suggest^38^ that exposure to PM is associated with attention deficits;^51^^,^^52^ specifically, PM_2.5_ was a risk factor for attentional/executive functions at ages 6–11 years, especially for girls.^52^ Exposure to PM_2.5_ was also associated with decreased learning and memory function and a higher risk of developing learning disabilities in boys. Additionally, one study observed evidence that PM_2.5_ was detrimental to executive function skills, and PM_2.5_ during commuting was associated with reduced growth in working memory.^53^ In fact, PM_2.5_ seems to be an air pollutant associated with adverse central nervous system (CNS) outcomes^54^ and has the most detrimental effects in comparison to other air pollutants such as NO_2_ and O_3_.^55^

Excessive exposure to metal has a detrimental effect on the nervous system. Neurons and glia in the developing brain are vulnerable to damage from metals such as lead and mercury, which can result in permanent neurodevelopmental damage.^56^ Lead is the metal best known for affecting cognitive health in children and causing behavioral disorders.^56^ High levels of lead exposure are associated with higher odds of having ADHD,^57^ loss of brain volume in the prefrontal cortex, and lower levels of gray matter^57^ and have been referenced as one of the causes of ASD development.^56^ Environmental exposure to mercury increases the chances of ASD and ADHD,^57^ and this exposure can lead to neuroinflammation, dendritic growth, and mitochondrial dysfunction (Table 1).

**Table 1:** Pollutants, sources and impact on health.

Classes	Pollutants	Pollutant source	Impact on health
Gases	SO_2_	Compounds secondary to chemical reactions, burning of fuels containing sulfur (diesel). Means of transport (that use oil burning)	Respiratory tracts: Highly soluble: they extensively affect the upper airways and skin.
	NO_2_	Thermal energy generating stations. Motor vehicle exhaust. Planes, trains, ships and the like. Forklifts and gas stoves.	Respiratory tracts: Less soluble: they penetrate deeper into the lung. It is related to the development of asthma, increased risk of exacerbations, and emergency room visits for asthma. Increased likelihood of acute respiratory infections. Exposure to NO_2_ is also associated with the development of atopy, wheezing, and lower FEV1. Central nervous system Worse neurological development. Cardiovascular system: Association with high blood pressure and increased prevalence and significantly associated with the risk of childhood obesity and higher BMI (specifically, the likelihood increases when the exposed child is already obese).
	O_3_	Photocopiers, room air purifiers, disinfection devices and laser printers	Respiratory tracts: Less soluble: they penetrate deeper into the lung. Exposure is associated with effects such as decreased peak expiratory flow, low FEV1 in children, and increased asthma-related emergency room visits. Cardiovascular system: Significantly associated with high blood pressure and 10 μg/m^3^ increases in O_3_ exposure are associated with an increased risk of obesity.
	CO	Motor vehicle (especially when indoors - garages and tunnels). Home heating systems (especially if malfunctioning), ovens, wood stoves, ice resurfacing equipment.	Respiratory tracts: Highly soluble and non-irritating, passing rapidly into the bloodstream, its toxicity results from the successful competition with oxygen for binding to hemoglobin, resulting in tissue hypoxia.

Classes	Pollutants	Source of the pollutant	Impact on health

Particulate matter (PM)	PM_0.1_ PM_2.5_ PM_10_	- Fuel burning - Incineration of industrial waste, - Wood stoves and the like - Pollens and other bioaerosols; - Coarse dust from dirt roads - Surface abrasion of tires - Construction waste (coarse particles)	Respiratory tracts: 1) Larger particles (PM_10_) are retained in the trachea, bronchi, and bronchioles. The fine particles (PM_2.5_) reach the pulmonary alveoli and are captured by the cells and transported to the bloodstream. Ultrafine particles (PM_0.1_) pass easily through the alveolar-capillary membrane and therefore have greater systemic toxicity. 2) Risk of emergency room visits and hospitalizations related to asthma exacerbations. PM_2.5_ was associated with wheezing episodes in children aged 2 to 10 years. PM_2.5_ and PM_10_ were related to the prevalence of childhood allergic rhinitis and visits to the emergency room and hospitalizations for respiratory infections. 3) Prolonged exposures can lead to local inflammation and fibrosis and predispose to lung carcinoma. Central nervous system: PM_2.5_ was a risk factor for attention/executive functions at ages 6 to 11 years, especially in girls. It is associated with decreased learning and memory in boys. Additionally, PM_2.5_ was detrimental to executive function skills and was associated with reduced growth in working memory. Cardiovascular system: PM contributes significantly to cardiovascular toxicity and increases the risk of developing diseases. Association between PM_2.5_ and PM_10_ exposure with high blood pressure in children. Exposure to PM_1_ and PM_2.5_ are associated with higher chances of childhood obesity and increased BMI.
Compounds carried by PM – heavy metals	Arsenic	Biomass – Charcoal / Wood	Respiratory tracts: In addition to invading organs and causing direct damage, the presence of inflammatory cells in the airways (polymorphonuclear leukocytes) and inflammatory cytokines in bronchoalveolar lavage fluid increase. Increased levels of nitric oxide fraction in asthmatics with multisystem effects. Central nervous system: Neurons and glia in the developing brain are vulnerable to damage caused by metals such as lead and mercury, which can result in permanent neurodevelopmental damage. Lead is the metal best known for affecting cognitive health in children and causing behavioral disorders. High levels of lead exposure are associated with higher likelihood of having ADHD, loss of brain volume in the prefrontal cortex, and lower levels of gray matter and have been referenced as one of the causes of ASD development. Significant associations were found between exposure to lead, inorganic arsenic, and cadmium and arterial hypertension in childhood Cardiovascular system: Significant associations were found between exposure to lead, inorganic arsenic, and cadmium and arterial hypertension in childhood.
	Lead	Biomass – Charcoal / Wood	
	Cadmium	Biomass – Charcoal / Wood	
	Sulphuric acid	Biomass – Charcoal / Wood	
	Aromatic hydrocarbons	Biomass – Charcoal / Wood	
	Diesel Emission Particulates (DEPs)	Burning of petroleum products	

## Recommended measures

Health professionals are reliable sources of information and advice; they play a very important role not only in treating health problems caused by air pollution but also in educating family members and patients about risks and solutions, as well as communicating with the general public and government leaders.

Health workers should increase their role in managing children's exposure to air pollution with better methods of care, prevention, and collective action. Thus, they should:

• Be informed: Understand air pollution as a risk factor for people; identify the sources of environmental exposure in the communities where they work.
• Recognize medical conditions associated with or related to exposure: A healthcare provider can identify risk factors related to air pollution by asking pertinent questions about the environments where their patients live or work.
• Investigate, publish, and disseminate knowledge: health professionals can conduct research on the health effects of air pollution and publish the results of studies on the causes, and communication strategies for social and behavioral change.
• Prescribe solutions and educate families and communities: For problems related to air pollution, such as the consumption of fuels and appliances that consume less energy, health professionals can play a role. Train others in the field of health and education: Health professionals can and should increase the scope of their messages on the health risks of air pollution and strategies to reduce them. They can engage their colleagues in
• Their workplaces, local health centers, conferences, and professional associations. They can support the inclusion of children's environmental health in the curriculum of elementary and higher education institutions, particularly in medical and nursing schools.
• Advise on solutions for political representatives and leaders from other sectors: Health professionals are well placed to share their knowledge with decision-makers, including members of local governments and school boards, and with other community leaders. Health workers can faithfully convey the health burden caused by air pollution to leaders, support better standards and policies to reduce harmful exposure, advocate for monitoring, and emphasize the need to protect vulnerable people.

## Conclusions

The evidence highlights the profound impact of air quality on the health of children and adolescents. From respiratory disorders to neurodevelopmental impairment, air pollution remains a widespread threat, particularly for vulnerable populations. Immediate actions at political, community, and individual levels are required to mitigate these risks. Future research should focus on figuring out mechanisms of tissue damage and evaluating the effectiveness of interventions, mechanisms, and effects of environmental exposure, as well as developing possible treatments, prevention, and management.

## Conflicts of interest

The authors declare no conflicts of interest.
